# Performance in Omics Analyses of Blood Samples in Long-Term Storage: Opportunities for the Exploitation of Existing Biobanks in Environmental Health Research

**DOI:** 10.1289/ehp.1205657

**Published:** 2013-02-05

**Authors:** Dennie G.A.J. Hebels, Panagiotis Georgiadis, Hector C. Keun, Toby J. Athersuch, Paolo Vineis, Roel Vermeulen, Lützen Portengen, Ingvar A. Bergdahl, Göran Hallmans, Domenico Palli, Benedetta Bendinelli, Vittorio Krogh, Rosario Tumino, Carlotta Sacerdote, Salvatore Panico, Jos C.S. Kleinjans, Theo M.C.M. de Kok, Martyn T. Smith, Soterios A. Kyrtopoulos, on behalf of the EnviroGenomarkers Project Consortium

**Affiliations:** 1 Department of Toxicogenomics, Maastricht University, Maastricht, the Netherlands; 2 Institute of Biology, Medicinal Chemistry and Biotechnology, National Hellenic Research Foundation, Athens, Greece; 3 Department of Epidemiology and Biostatistics, School of Public Health, Faculty of Medicine, MRC-HPA Centre for Environment and Health, Imperial College London, London, United Kingdom; 4 Department of Surgery and Cancer, Faculty of Medicine, Biomolecular Medicine, Imperial College London, London, United Kingdom; 5 Institute for Risk Assessment Sciences, Division of Environmental Epidemiology, Utrecht University, Utrecht, the Netherlands; 6 Department of Public Health and Clinical Medicine, and Department of Biobank Research, Occupational and Environmental Medicine, Umeå University, Umeå, Sweden; 7 Nutrition Research, Department of Public Health and Clinical Medicine, and Department of Biobank Research, Umeå University, Umeå, Sweden; 8 The Institute for Cancer Research and Prevention, Florence, Italy; 9 Epidemiology and Prevention Unit, Fondazione IRCCS Istituto Nazionale dei Tumori, Milan, Italy; 10 Cancer Registry and Histopathology Unit, “Civile–M.P. Arezzo” Hospital, Ragusa, Italy; 11 Center for Cancer Prevention (CPO-Piemonte), Turin, Italy; 12 Unit of Epidemiology and Molecular Genetics, Human Genetics Foundation (HuGeF), Turin, Italy; 13 Department of Clinical and Experimental Medicine, Federico II University, Naples, Italy; 14 Genes and Environment Laboratory, School of Public Health, University of California, Berkeley, Berkeley, California, USA

**Keywords:** biomarkers, epigenomics, metabolomics, metabonomics, molecular epidemiology, proteomics, transcriptomics

## Abstract

Background: The suitability for omic analysis of biosamples collected in previous decades and currently stored in biobanks is unknown.

Objectives: We evaluated the influence of handling and storage conditions of blood-derived biosamples on transcriptomic, epigenomic (CpG methylation), plasma metabolomic [UPLC-ToFMS (ultra performance liquid chromatography–time-of-flight mass spectrometry)], and wide-target proteomic profiles.

Methods: We collected fresh blood samples without RNA preservative in heparin, EDTA, or citrate and held them at room temperature for ≤ 24 hr before fractionating them into buffy coat, erythrocytes, and plasma and freezing the fractions at –80^o^C or in liquid nitrogen. We developed methodology for isolating RNA from the buffy coats and conducted omic analyses. Finally, we analyzed analogous samples from the EPIC-Italy and Northern Sweden Health and Disease Study biobanks.

Results: Microarray-quality RNA could be isolated from buffy coats (including most biobank samples) that had been frozen within 8 hr of blood collection by thawing the samples in RNA preservative. Different anticoagulants influenced the metabolomic, proteomic, and to a lesser extent transcriptomic profiles. Transcriptomic profiles were most affected by the delay (as little as 2 hr) before blood fractionation, whereas storage temperature had minimal impact. Effects on metabolomic and proteomic profiles were noted in samples processed ≥ 8 hr after collection, but no effects were due to storage temperature. None of the variables examined significantly influenced the epigenomic profiles. No systematic influence of time-in-storage was observed in samples stored over a period of 13–17 years.

Conclusions: Most samples currently stored in biobanks are amenable to meaningful omics analysis, provided that they satisfy collection and storage criteria defined in this study.

The use of omics technologies has improved our understanding of the mechanisms of toxicity and led to valuable new knowledge for environmental health research (Ellinger-Ziegelbauer 2009; McHale et al. 2010). By providing global and quantitative information on changes in critical cellular components under the influence of environmental factors, omics profiling greatly facilitates the discovery of biomarkers and is seen as a key tool in the development of the concept of the exposome (Rappaport and Smith 2010).

The application of omics technologies in epidemiological studies raises certain practical issues of sample suitability, especially in relation to RNA quality for transcriptomics analysis, requiring that care be taken for blood samples to be collected and stored in the presence of RNA preservative. However, millions of human biosamples currently in cold storage in older biobanks were collected and processed by methods that did not anticipate the demands of omics technologies. Those biobanks represent a precious resource for environmental health research, especially in view of the fact that newly constructed biobanks will take many years to accrue enough cases of chronic diseases in their prospective cohorts to allow relevant biomarker research. Yet no study has evaluated systematically the influence on omic profiles of the handling and prolonged storage of blood samples and their components in these biobanks.

In the context of the European project EnviroGenomarkers (http://www.envirogenomarkers.net), blood-derived biobank samples are being analyzed on multiple omic platforms with the aim of discovering new biomarkers of exposure and disease risk. As a first step in this project, we evaluated the reliability of omics data obtained from archived biosamples collected before the advent of omics technologies.

## Materials and Methods

The omics technologies we used include transcriptomics, epigenomics (CpG methylation), and plasma ultra performance liquid chromatography–time-of-flight mass spectrometry (UPLC-ToFMS) metabolomics. In addition, we used a multianalyte profiling platform as a tool for a wide-target plasma proteomics screen. We complied with all international regulations regarding the use of human participants. The research ethic committees of the University of Maastricht and of the National Hellenic Research Foundation approved the use of volunteers, and written informed consent was obtained from all volunteers prior to the study. The corresponding ethical committees approved the use of biobank samples.

During phase 1 of the study, we established methods for the isolation of RNA of the desired quality from buffy coats isolated from blood freshly collected and processed without RNA preservative. We also evaluated the influence on omics profiles of sample handling and storage-related parameters selected after scrutiny of the procedures employed at the biobanks participating in the study. The results obtained were used to establish minimum criteria that samples must satisfy in order to be suitable for reliable omics analysis. In order to evaluate the influence of long-term storage, during phase 2 we analyzed historic samples that satisfied these criteria. The samples had been stored in the European Prospective Investigation into Cancer and Nutrition (EPIC)-Italy and the North Sweden Health and Disease Study (NSHDS) biobanks (Bingham and Riboli 2004; Hallmans et al. 2003).

***Sample collection.* Phase 1.** We collected fresh blood from healthy volunteers using three different anticoagulants (heparin, EDTA, and citrate) and processed the blood in different ways. For practical reasons we conducted several blood collection experiments, in the context of which different variables were evaluated [for details, see Supplemental Material, pp. 6–7 (http://dx.doi.org/10.1289/ehp.1205657)]. After allowing the blood samples to stand at room temperature for various times ≤ 24 hr (“bench time”), we separated buffy coats, erythrocytes, and plasma by centrifugation for 15 min at 1,500*g* at room temperature, followed by aliquoting and immediate storage of the fractions at –80^o^C or in liquid nitrogen. To control for effects of interindividual variation, in one experiment we collected blood from one person in each of the three anticoagulants, processed it for fractionation, and stored the fractions both at –80^o^C and in liquid nitrogen but without variation in bench time.

The duration of cold storage of the blood fractions prior to omics analysis varied from several weeks to several months. We conducted full-scale metabolomics and wide-target proteomics analysis on all samples from a single blood collection experiment, in the context of which we evaluated all combinations of the parameters of interest (donors, bench times, anticoagulants, storage temperature). On the other hand, for practical reasons we generally conducted transcriptomics and epigenomics analyses aimed at evaluating the influence of individual variables on a more limited number of samples.

**Phase 2.** We used biosamples from the participating biobanks, satisfying the cut-off criteria established during phase 1, to evaluate the quality of extracted RNA and DNA and carry out omics analyses. Samples from EPIC-Italy contained citrate as anticoagulant and had been stored in cryostraws in liquid nitrogen for 11–19 years. Their recorded collection-to-storage times were 55–347 min. Samples from NSHDS contained heparin or EDTA as anticoagulant and had been stored in plastic cryovials at –80^o^C for 4–19 years. Their collection-to-storage time was always < 1 hr. To evaluate the impact of storage time on the different omics profiles, we analyzed samples from the same set of 31 subjects from each biobank. To minimize the effect of variables other than storage time, these samples were selected to come only from healthy female donors and from the same collection center per biobank. Their storage time prior to analysis was 13–17 years, and the collection-to-storage times for the EPIC-Italy subset ranged from 100 to 198 min.

*RNA and DNA isolation*. To establish methods for RNA extraction from buffy coats stored in the absence of RNA preservative, we allowed phase 1 samples to thaw while fully immersed in RNAlater or Qiazol (QIAGEN, Venlo, the Netherlands) and subsequently extracted RNA according to the manufacturer’s instructions. We quantified RNA with a Nanodrop ND-1000 spectrophotometer (Thermo Scientific, Wilmington, DE, USA) and used an Agilent 2100 Bioanalyzer (Agilent Technologies, Amstelveen, the Netherlands) to assess its quality, including its RNA integrity number (RIN), which represents the degree of RNA fragmentation (Schroeder et al. 2006). All RINvalues were > 6, as required for good quality microarray-based analysis. Although the above procedures also allow extraction of microRNA (miRNA), this was not systematically assessed in these samples.

In phase 2, we adapted the RNA extraction methodology developed in phase 1 for use with biobank samples. We handled all samples individually and immediately after retrieval from storage. We divided sample-containing cryostraws from EPIC-Italy for different applications by cutting them with RNase-free tools on a stainless steel plate imbedded in a box of dry ice to prevent thawing during handling. Then we pushed out half of the frozen buffy coat with a thin stainless steel plunger directly into 1.2 mL of the RNAlater (QIAGEN) solution. The other half was used later for DNA isolation. We retrieved NSHDS samples from their cryovials in a frozen state by making a small opening at the bottom of each vial using a hot plunger and pushing the sample out with another plunger. After subdividing the buffy coat on a dry ice–cooled steel plate using an RNase-free scalpel, we immediately thawed the part destined for RNA extraction in 1.2 mL of RNAlater (QIAGEN) [see Supplemental Material for a video of these procedures (http://dx.doi.org/10.1289/ehp.1205657)]. We replaced the remaining pellet in a new cryovial and immediately returned it to cold storage for later DNA isolation. RNA was isolated on the same day with the RiboPure™ Blood kit (Ambion, Austin, TX, USA) using the manufacturer’s miRNA isolation protocol.

We used buffy coats free of RNA preservative for DNA isolation because material thawed in the presence of RNAlater or Qiazol (QIAGEN) proved impossible to dissolve for DNA isolation. We thawed the samples on ice and isolated DNA using the QIAamp Blood Mini Kit (QIAGEN), evaluating it spectrophotometrically and by agarose gel electrophoresis.

*Transcriptomics*. We conducted Agilent 4×44K human whole genome microarray analyses by standard methodology. Briefly, we reverse transcribed each RNA sample into cDNA and labeled it with cyanine 3 prior to hybridization. Subsequently, we washed the slides and scanned them using an Agilent Technologies G2565CA DNA Microarray Scanner. We established the technical performance and quality of the microarrays by visual evaluation of the scan images before and after within- and between-array normalization (using LOESS and A-quantile, respectively). We imputed missing values in GenePattern (version 3.1; Broad Institute, MIT and Harvard University, Cambridge and Boston, MA, USA) using the *k* nearest neighbors approach (*k* = 15, Euclidian metric). [For more details on the transcriptomics and other omics methodologies employed, see Supplemental Material, pp. 6–11 (http://dx.doi.org/10.1289/ehp.1205657).]

*Epigenomics*. We conducted genome-wide analysis of DNA methylation using Infinium HumanMethylation450 BeadChips (Illumina, San Diego, CA, USA), which contain 485,764 probes (> 99% with CpG dinucleotides), following the manufacturer’s recommendations. We preprocessed the data with the GenomeStudio (version 2011.1) Methylation module (version 1.9; Illumina) and evaluated them using an adaptation of HumMeth27QCReport (Mancuso et al. 2011). We used Gene ARMADA (Chatziioannou et al. 2009) for within- and between-array normalization (linear LOESS and A-quantile, respectively) and imputation of missing values (*k* nearest neighbors approach).

*Metabolomics*. We analyzed plasma samples by UPLC-ToFMS after deproteinization with methanol. We conducted reverse-phase chromatography on an Acquity UPLC system (Waters Corporation, Milford, MA, USA) with a C_18_ column (Waters) and binary gradient elution (20–100% acetonitrile/water for ~ 25 min). Online analysis of the eluent was performed using a quadrupole time-of-flight mass spectrometer (QTOF-MS; Waters), with data collected in centroid mode in the 100–1,000 *m/z* range. In phase 2, we prepared samples in batches by biobank. Data were processed using Databridge and XCMS software (Waters).

*Wide-target plasma proteomics*. We conducted targeted proteomic analysis of plasma samples using the Lab-MAP multianalyte profiling technology (Luminex, Austin, TX, USA). We analyzed phase 1 samples for interleukin (IL)2, IL6, IL8, IL10, and tumor necrosis factor-α (TNFα) as previously described (Saberi-Hosnijeh et al. 2010), and we analyzed phase 2 samples for an additional 23 proteins related to immune responses [for a full list, see Supplemental Material, pp. 10–11, (http://dx.doi.org/10.1289/ehp.1205657)] according to the manufacturer’s protocol. Phase1 and 2 samples were run in a single batch on a single plate. Nondetectable concentrations (< 1.22 pg/mL for all analytes) were imputed via a maximum likelihood estimation method (Lubin et al. 2004).

*Statistical evaluation*. The data were evaluated using principal component analysis (PCA), analysis of variance (ANOVA), paired *t*-test, mixed effect models, relative standard deviation (RSD = SD/mean), false discovery rate (FDR; Storey’s *q*-value), and short time–series expression miner (STEM) analysis (Ernst and Bar-Joseph 2006). PCA plots were used to visualize the impact of different sample handling parameters on omics signals as reflected in the variation of the different principal components. STEM analysis allows the identification of significant temporal trends in expression profiles and the genes associated with them. Because of the severe heteroscedasticity of β-values (representing the fractional methylation at any given site) at highly methylated or unmethylated CpG sites, M values [M = log_2_(methylated/unmethylated)] were used for the statistical analysis of DNA methylation data (Du et al. 2010).

## Results

*Transcriptomics*. Phase 1. RNA quality and quantity were both significantly (*p* < 1 × 10^–5^) higher in buffy coat samples thawed in the presence of RNAlater as compared with Qiazol (RIN: 7.17 ± 0.51 vs. 6.14 ± 0.72; RNA yield: 6.03 ± 2.16 vs. 2.25 ± 1.04 μg), and for this reason the former was employed routinely. No systematic effect of bench time, anticoagulant, or storage temperature on RIN values was observed (Table 1). RNA yield was unaffected by bench time and was higher for citrate samples regardless of storage temperature (*p* < 0.01, possibly due to minor interference of heparin and EDTA in the RNA extraction procedure) and for –80^o^C samples regardless of anticoagulant (*p* < 0.05). We confirmed these findings using blood samples originating from one subject collected with different anticoagulants and a bench time of 0 hr (results not shown).

**Table 1:** RINs and RNA yields (μg) of fresh samples from four subjects (mean ± SD) according to anticoagulant, storage temperature, and bench time (0–24 hr).

Anticoagulant, storage temperature	0 hr		2 hr		4 hr		8 hr		24 hr	
	RIN	RNA yield	RIN	RNA yield	RIN	RNA yield	RIN	RNA yield	RIN	RNA yield
Citrate										
–80oC	7.15 ± 0.14	8.27 ± 2.23	7.25 ± 0.42	8.90 ± 0.53	7.43 ± 0.53	8.75 ± 1.59	7.70 ± 0.21	8.29 ± 1.64	7.38 ± 0.24	12.19 ± 4.93
Liquid nitrogen	7.10 ± 0.49	5.96 ± 1.43	7.68 ± 0.11	5.74 ± 0.80	7.65 ± 0.14	4.77 ± 0.56	7.33 ± 0.11	6.56 ± 2.77	7.08 ± 0.04	6.62 ± 3.55
EDTA										
–80oC	6.43 ± 0.88	5.14 ± 2.20	6.53 ± 0.88	10.46 ± 7.33	7.30 ± 0.21	5.21 ± 2.93	7.50 ± 0.00	6.98 ± 7.06	7.60 ± 0.35	5.50 ± 3.17
Liquid nitrogen	6.75 ± 0.64	3.29 ± 1.26	6.95 ± 0.92	4.09 ± 2.61	7.55 ± 0.07	4.24 ± 3.05	7.18 ± 0.04	3.95 ± 1.80	7.20 ± 0.14	5.22 ± 1.16
Heparin										
–80oC	6.95 ± 0.21	4.81 ± 0.43	5.28 ± 2.93	6.48 ± 5.23	7.50 ± 0.00	7.06 ± 4.14	6.78 ± 1.31	6.70 ± 3.15	7.93 ± 0.18	6.51 ± 1.60
Liquid nitrogen	6.68 ± 0.95	3.20 ± 0.97	6.88 ± 0.95	4.24 ± 2.17	7.53 ± 0.18	3.93 ± 2.62	7.45 ± 0.35	4.09 ± 0.15	7.50 ± 0.00	3.73 ± 0.99
Yields were obtained from 0.4–0.5 mL of buffy coat (corresponding to ~ 2 mL blood).										

**Figure 1:**
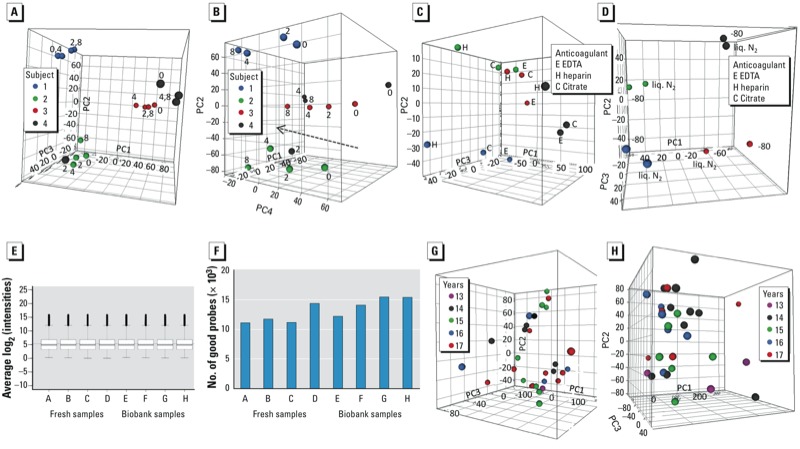
Transcriptomics. (*A*–*D*) Phase 1: data from four different subjects (samples from same subjects are indicated with same symbols). (*A*) PCA plot on samples with different bench times (indicated in hours by the symbol labels) (EDTA, –80 ^o^C; proportion of variance explained: PC1, 31%; PC2, 24%; PC3, 14%). (*B*) PCA on same samples but using PC4 instead of PC3 (proportion of variance explained: PC4, 10%); the line indicates the bench time–related trend. (*C*) PCA on samples with different anticoagulants (bench time 2.5 hr, –80^o^C; proportion of variance explained: PC1, 57%; PC2, 13%; PC3, 10%). (*D*) PCA on samples with different storage temperatures (EDTA, bench time 0 hr; proportion of variance explained: PC1, 39%; PC2, 21%; PC3, 19%); liq. N_2_, liquid nitrogen. (*E*–*H*) Phase 2: (*E*,*F*) Comparison of microarray data from four fresh and four biobank samples. (*E*) Box plots of the average intensity levels after LOESS and A-quantile normalization; boxes correspond to the 25th and 75th percentiles, whiskers indicate minimum and maximum values, and circles represent outliers. (*F*) Numbers of good array probes. (*G*,*H*) PCA plots on storage time in biobank; the legend indicates the number of years in storage. (*G*) EPIC-Italy (proportion of variance explained: PC1, 32%; PC2, 13%; PC3, 8%). (*H*) NSHDS (proportion of variance explained: PC1, 40%; PC2, 10%; PC3, 8%).

We performed transcriptomics analysis of the effects of donor and bench time on material from four subjects. Genes with more than one flagged/missing time point for any subject were completely filtered out of the data set, leaving 27,181 genes. Plots of principal components (PC) according to the various sample-related parameters (Figure 1A,B) showed clear separation between the subjects (except for one time point of one subject), based on PC1–3, whereas a bench time–dependent trend was observed ≤ 8 hr in PC4 [the bench time of 24 hr was omitted because a small-scale RT-PCR experiment had already shown substantial gene expression changes at this time point (results not shown)]. We further investigated this trend by performing an ANOVA across the four time points and using the resulting 3,372 significant genes (*p* < 0.05) in a STEM analysis to identify significantly represented temporal gene expression profiles. Two significant profiles were identified, corresponding to a gradual decrease or increase in expression and together accounting for 83% of the genes with significant differences in expression based on ANOVA, with a between-subject of overlap of 90% [see Supplemental Material, Figure S1A,B (http://dx.doi.org/10.1289/ehp.1205657)]. Time-point comparisons showed considerable numbers of differentially expressed genes (1,000–3,000) at all time points, their numbers roughly doubling in going from 2 hr to 4 hr (see Supplemental Material, Figure S1C). A pathway analysis of the two significant temporal STEM profiles revealed mainly involvement of the biological processes apoptosis, stress signaling, and DNA damage repair (results not shown). A list of genes with significant differences in expression based on ANOVA (Bonferroni-corrected *p* < 0.05) that may be suitable as bench time effect markers is presented in Supplemental Material, Table S1.

For the anticoagulant and storage temperature analyses, again on groups of four subjects, all genes flagged in any subject were filtered out, leaving 28,478 and 27,552 genes, respectively. PCA showed a clear separation between subjects, but also some distance between the three anticoagulants, especially heparin (Figure 1C). Paired *t*-test analysis showed significant differences between all three anticoagulants [see Supplemental Material, Figure S1D (http://dx.doi.org/10.1289/ehp.1205657)], with the largest differences (although not as large as with bench time) being found between heparin and either EDTA or citrate, both with and without a log_2_ ratio cut-off of 0.5. We also identified differences in the gene expression pattern between samples stored at –80^o^C and in liquid nitrogen (Figure 1D), with 2,193 differentially expressed genes (551 genes with an additional 0.5log_2_ ratio cut-off), but the FDR stayed relatively high (35%).

To compare the impact of sample processing–related variables to the impact due to assay technical variability, we used technical repeats (2–3 repeats per subject) to determine the coefficient of variation of corresponding log_2_-expression signals (average of 2.7%). Same-individual bench time variation for all but one time point comparison (4 hr vs. 8 hr) and for EDTA versus heparin was significantly higher than technical variation (ranging ≤ 4.2%), whereas the variation was not significantly different for the other anticoagulant and storage temperature comparisons. This means that bench time is the main source of sample processing–related variability, although any effects of the other two variables may be overshadowed by technical noise.

**Phase 2.** Using the procedures described, adequate amounts of RNA with RIN > 6.0 (average RIN = 7. 2, similar to fresh phase 1 samples) could be isolated from approximately 85% of the extracted biobank samples (64 from EPIC-Italy and 50 from NSHDS) (Table 2), with no observable systematic effect of storage time (results not shown).

**Table 2:** Average RINs and RNA yield (μg) from biobank samples.

Cohort	n	Percent RIN > 6	RIN (average ± SD)	RNA yield (mean ± SD)
EPIC-Italy	64	95	7.1 ± 0.7	3.9 ± 1.7
NSHDS	50	92	7.4 ± 0.9	12.2 ± 7.5
The EPIC-Italy sample set included six samples stored at –80oC with a RIN of 6.8 ± 0.5 and RNA yield of 5.1 ± 1.2. The remaining samples were stored in liquid nitrogen. The NSHDS sample set included nine samples with EDTA as anticoagulant with a RIN of 6.7 ± 0.8 and RNA yield of 13.9 ± 6.8 μg. The remaining samples used heparin. EPIC-Italy and NSHDS yields were obtained from half a cryostraw or half a microcentrifuge tube of buffy coat, corresponding to ~ 0.25 and 0.7–1.0 mL buffy coat (corresponding to ~ 3 and ~ 9 mL blood), respectively.				

To test the performance of biobank samples in transcriptomics analysis, we initially used four EPIC-Italy samples to compare the technical quality of the microarray data with those obtained with four phase 1 samples stored at –80°C (different donors, two heparin and one EDTA with a bench time of 0 hr and one heparin with a bench time of 24 hr). All RNAs were hybridized against freshly isolated RNA from phase 1 samples. No differences could be seen between the quality of the arrays hybridized with fresh or biobank samples. After normalization, a box plot showed similar data distribution between all samples (equal medians) (Figure 1E). After filtering flagged features, we observed no significant difference in the number of remaining high-quality probes across the arrays (Figure 1F).

PCA of the transcriptomic profiles of 31 samples from each biobank, selected as described in “Materials and Methods,” does not suggest any consistent storage time effect within the range of 13–17 years (Figure 1G,H). ANOVA across these samples showed only 14 and 76 genes for EPIC-Italy and NSHDS, respectively, (of a total of 29,662) to vary significantly (*p* < 0.0033) according to storage time; however, the FDR level was around 100%, meaning that these were most likely false positives. We could not make a meaningful evaluation of the effect of collection-to-storage time on the transcriptomic (or any other) profile because of the small range of variation of this variable among the analyzed samples (100–198 min).

A comparison of six low-RIN samples (RIN range, 5.9–6.9) with six high-RIN samples (RIN range, 8.5–8.8) yielded only one differentially expressed gene at an FDR of 10% (results not shown), indicating that RNA quality was not a significant factor influencing the transcriptomic profiles of biobank samples. As an additional test of data quality, we evaluated the expression of three blood reference genes [beta-2 microglobulin (*B2M*); glyceraldehyde-3-phosphate dehydrogenase (*GAPDH*); and protein phosphatase 1, catalytic subunit, alpha isozyme (*PPP1CA*)] and 11 immunomodulatory marker genes [chemokine (C-X-C motif) ligand 1 (melanoma growth stimulating activity, alpha; *CXCL1*); heme oxygenase (decycling) 1 (*HMOX1*); intercellular adhesion molecule 1 (*ICAM1*); IL-1, beta (*IL1B*); IL-1 receptor antagonist (*IL1RN*); IL-6 receptor (*IL6R*); matrix metallopeptidase 9 (*MMP9*); prostaglandin-endoperoxide synthase 2 (*PTGS2*); serpin peptidase inhibitor, clade E, member 1 (*SERPINE1*); transforming growth factor, beta 1 (*TGFB1*); and *TNF*] (Karlovich et al. 2009) in these and in phase 1 samples (all bench times, four subjects) [see Supplemental Material, Table S2 (http://dx.doi.org/10.1289/ehp.1205657)]. All genes were expressed in all sample sets, with the log_2_-transformed intensities of the three reference genes and the majority of the immunomodulatory genes being > 10, statistically significantly higher than the average expression of all genes (*t*-test *p* < 0.01). These results support the absence of any major effect of long-term storage.

**Figure 2:**
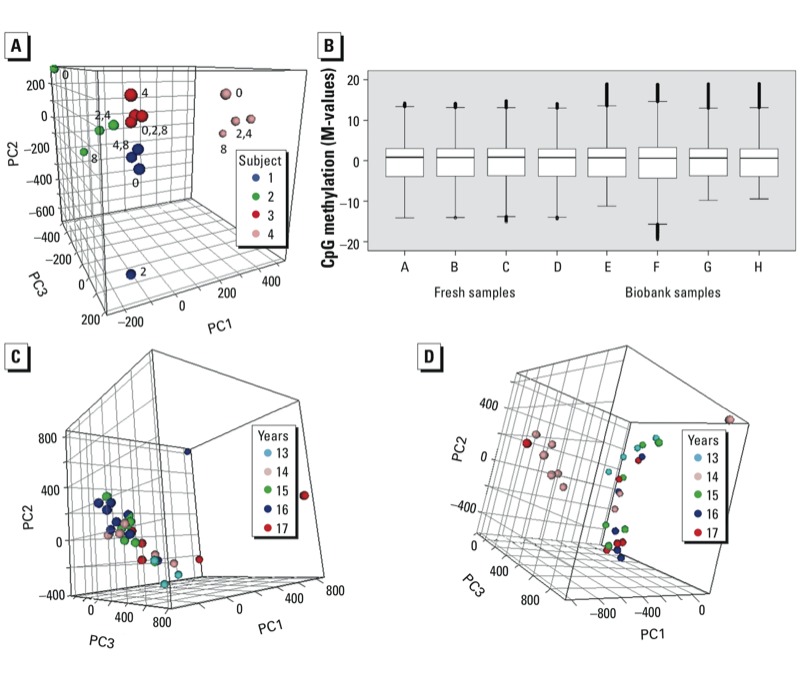
Epigenomics. (*A*) Phase 1, PCA plot on bench times from four subjects (samples from same subjects are indicated by same symbols, labels indicate bench time in hours) (EDTA, –80^o^C; proportion of variance explained: PC1, 37%; PC2, 18%; PC3 16%). (*B*) Comparison of methylation microarray data from four fresh and four biobank samples. Box plots of the M-values after LOESS and A-quantile normalization; boxes correspond to the 25th and 75th percentiles, whiskers indicate minimum and maximum values, and circles represent outliers. (*C*,*D*) Phase 2, PCA plots on storage time (number of years) in biobank [(EPIC Italy; proportion of variance explained: PC1, 20%; PC2, 10%; PC3, 7% (*C*); NSHDS, PC1, 19%; PC2 13%; PC3 6% (*D*)].

***Epigenomics*. Phase 1.** We did not find any effect of anticoagulant or storage temperature on the yield or quality of isolated DNA or on CpG methylation levels (data not shown). We evaluated the effects of bench time using the buffy coats of four subjects. PCA based on M-values showed clear separation between the subjects (Figure 2A). However, in contrast to the corresponding transcriptomics result, no time-dependent trend was evident in PC1-3 (Figure 2A) or other PCs (not shown). The mean coefficient of variation between corresponding probes with 0.01 < β⊇< 0.99 (thus limited to avoid spurious variability at very low signal intensities) in a 0-hr versus 8-hr comparison was 12.2%, not significantly different from that between technical replicates (13.2%). In an ANOVA across the four time points, with an additional implementation of a threshold of 20% minimum variation in β, only 3,086 CpG sites (0.6% of the total) showed significant (*p* < 0.05) time-dependent variation. STEM analysis of this data set did not reveal a dominant time-pattern, and overlap between the four subjects was minimal (data not shown), strongly suggesting that this variation does not reflect a systematic cellular response.

**Phase 2.** DNA isolated from 42 EPIC-Italy and 38 NSHDS biobank samples was of good quality (260/280 optical density ratio, 1.75–1.85; molecular weight > 40,000 kD) and yields were comparable with those obtained with fresh material. We evaluated the suitability of this DNA for microarray-based analysis of CpG methylation by comparing four samples from EPIC-Italy and four samples from phase 1 buffy coats. The fraction of good probes was > 99.85% in all cases and only 0.069% of the probes had detection *p* > 0.05 in more than one sample and were thus completely excluded. Similar β-value distributions were observed in phase 1 and biobank samples (Figure 2B).

Although PCA of 31 samples from each biobank, stored for 13–17 years, showed some scatter [e.g., for samples collected in 1997 in EPIC-Italy and 1996 in NSHDS (13 and 14 years in storage, respectively)], no systematic trend was evident in relation to the storage time (Figure 2C,D). ANOVA results indicated that only 50 CpG sites in EPIC-Italy and 1 site in NSHDS samples showed significant variation (Bonferroni-adjusted *p* < 0.05) in methylation levels in relation to storage time.

**Figure 3:**
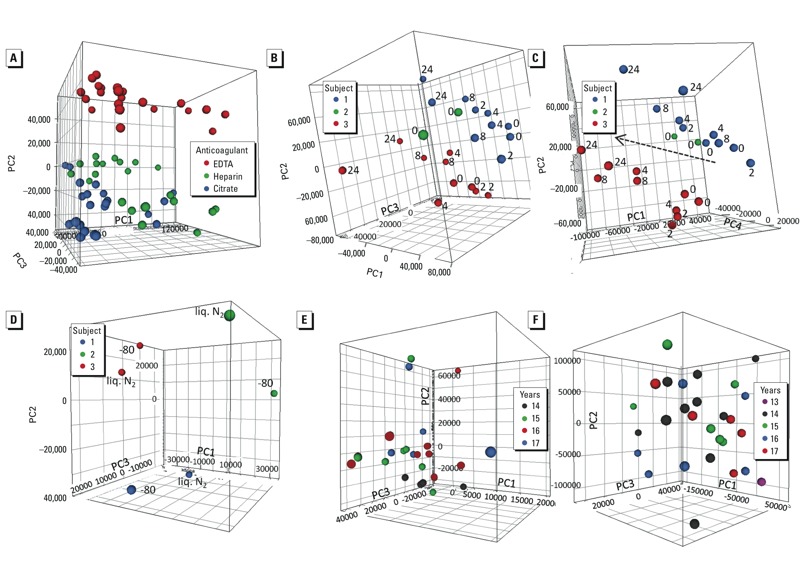
Metabolomics. (*A*–*D*) Phase 1. (*A*) PCA plot on anticoagulants from four subjects; because all samples were subjected to full metabolomics analyses, the points shown for each anticoagulant include different subjects, bench times, and storage temperatures (proportion of variance explained: PC1, 44%; PC2, 20%; PC3, 10%). (*B*) PCA on bench time for three subjects (different symbols denote different subjects and include two different storage temperatures per subject; the labels denote bench times in hours (only 0 hr bench time for one subject) (heparin; proportion of variance explained: PC1, 56%; PC2, 20% PC3, 12%). (*C*) PCA for same samples as *D* but PC4 instead of PC3 (proportion of variance explained: PC4, 6%); the line indicates the bench time–related trend. (*D*) PCA on storage temperature from four subjects (different symbols denote different subjects) (heparin, bench time 0 hr; proportion of variance explained: PC1, 51%; PC2, 31%; PC3, 14%); liq. N_2_, liquid nitrogen. (*E*–*F*) Phase 2. (*E*,*F*) PCA on storage time (years) in biobank [EPIC-Italy; proportion of variance explained: PC1, 35%; PC2, 10%; PC3, 8% (*E*); NSHDS; proportion of variance explained: PC1, 36%; PC2, 21%; PC3, 7% (*F*)].

***Metabolomics*. Phase 1.** Of the spectral features detected in all samples analyzed for different experimental conditions, 85.9% exhibited an RSD < 30% (median RSD = 13%) across the quality control samples, which consisted of identical aliquots of a pooled sample interspersed within the batch of regular samples [see Supplemental Material, pp. 9–10 (http://dx.doi.org/10.1289/ehp.1205657)]. A PCA plot based on these “robust” features indicated a clear separation according to anticoagulant regardless of donor and other variables (Figure 3A). For a given anticoagulant, the main sources of variation were the donors themselves and bench time [Figure 3B,C, heparin samples only (similar plots were obtained for EDTA and citrate plasma samples)], with the 8-hr and 24-hr time points separating away from the earlier time points. No general trend was observed in relation to the storage temperature (Figure 3D). The median RSDs of robust peaks reflecting variation by anticoagulant and subject were 11.7% and 18%, respectively, whereas the effect of bench time was much smaller, and that of storage temperature minimal (Table 3). The numbers of peaks that varied significantly (according to ANOVA) with both anticoagulant and bench time were substantially larger than expected by false discovery (71% of peaks at 2% FDR and 6% of peaks at 8% FDR, respectively), confirming the importance of these factors but also that bench time significantly affected only a relatively small number of metabolites. Similar analysis confirmed that the number of peaks affected by storage temperature (< 1%) was below that expected by false discovery.

**Table 3:** RSD (%) of metabolomics peaks across experimental conditions in phase 1.

Condition	P10	Median	P90
QC samples	7.0	13.0	37.0
Subjectsa	13.0	18.0	49.4
Anticoagulantsa,b	4.5	11.7	46.6
Storage temperaturesa,b	0.4	1.8	5.0
Bench timesa,b
0 vs. 2 hr	0.6	2.7	7.4
0 vs. 4 hr	1.0	2.8	7.1
0 vs. 8 hr	1.7	3.5	9.3
0 vs. 24 hr	2.2	4.8	15.4
P, percentile. RSDs were calculated by comparing samples differing in the condition indicated while keeping all other conditions constant; “QC samples” refers to comparison across identical quality control samples; “bench times” refers to comparison between samples with bench time 0 hr and the time indicated. aUsing only selected peaks with RSD < 30% across QCs. bData were normalized to the mean value of donor.			

**Phase 2.** To evaluate the effect of storage time, we analyzed samples from the same set of subjects as used for the other omics platforms (24 EPIC-Italy and 28 NSHDS plasma samples were available). PCA did not show any systematic effect of storage time (Figure 3E,F). Overall 77.2% (EPIC-Italy) and 72.4% (NSHDS) of spectral features exhibited an RSD of < 30% across the quality control samples. The variation of these robust features across all biobank samples was 2- to 3-fold greater than that associated with storage time (Table 4). ANOVA and false discovery analysis confirmed the absence of a statistically significant association between metabolite peaks and storage time.

**Table 4:** RSD (%) of metabolomics peaks across subjects and storage times for phase 2 samples.

Cohort	P10	Median	P90
EPIC-Italy			
All subjects	12.8	26.2	55.8
Storage time	3.9	10.8	24.3
NSHDS			
All subjects	13.8	27.8	54.6
Storage time	3.9	8.8	23.1
P, percentile. For each cohort, RSDs were calculated by comparing a) samples of all subjects, and b) the means of samples with the same storage time.			

**Figure 4:**
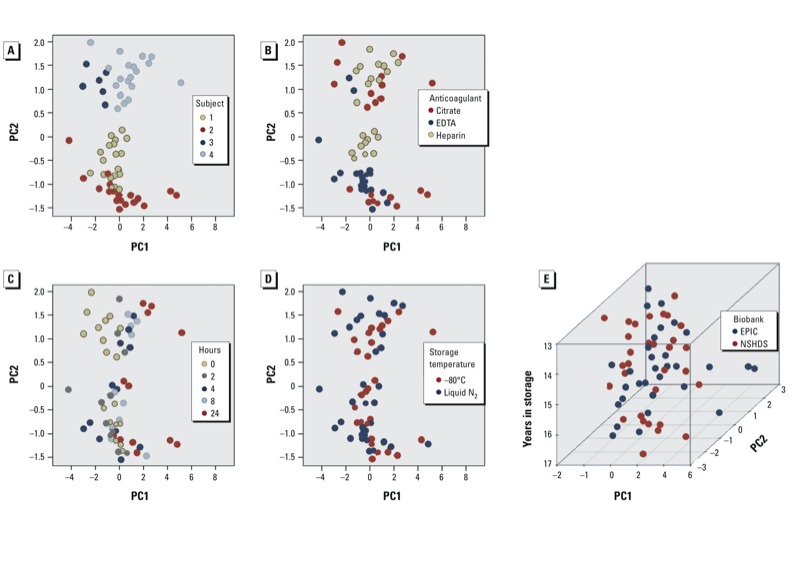
Proteomics. (*A*–*D*) Phase 1. PCA plot (proportion of variance explained: PC1, 56.4%; PC2, 25.0%) labeled for donor (*A*), storage temperature (*B*), bench times (*C*), anticoagulants (*D*). Because all samples were subjected to proteomic analyses, the points shown for each variable indicated include variation of the remaining variables. (*E*) PCA on storage time in the two biobanks.

***Wide-target plasma proteomics*. Phase 1.** Owing to the small number of features measured, only two significant principal components were observed. Figure 4A shows that the greatest variation was attributable to the donor, although separation was observed *a*) by anticoagulant (Figure 4B), with citrate resulting in higher levels of IL2 and IL6 and heparin resulting in higher levels of IL8 (results not shown), and *b*) by bench time (Figure 4C), with the 8-hr and 24-hr time points deviating the most from the earlier ones, which were relatively similar. No effect of storage temperature was observed (Figure 4D). The coefficient of variation between different anticoagulants (citrate vs. heparin, median 17%; EDTA vs. heparin, median –2.0%) was substantially larger than that between technical replicates (median 2.2%). The latter was similar to the coefficient of variation for the 0-hr versus 2-hr bench time comparison (median –3.0%) and comparable for most analytes to that for 0 hr versus 4 hr (median 10.5%; most variation being due to one outlying analyte). However, the variation was substantially increased for the 0 hr versus 8 hr comparison, and even more for the 0 hr versus 24 hr comparison, where it reached a median value of 77%.

**Phase 2.** PCA based on the same sets of 31 subjects from each biobank as used with the other platforms did not reveal any systematic effect of storage period (Figure 4E) or collection-to-storage time (data not shown), nor were any associations found with any of the individual analytes. The measured cytokine levels were in the same range as observed in phase 1 (results not shown), suggesting comparability between fresh and biobank samples.

## Discussion

We have evaluated the influence of collection and storage conditions of buffy coat and plasma on sample performance in a series of omics assays, using freshly collected samples as well as samples stored in biobanks for nearly two decades. The key findings can be summarized as follows.

*Transcriptomics.* Transcriptomics-quality RNA can, in general, be isolated from buffy coats frozen in the absence of RNA preservative by thawing the samples in the presence of RNAlater, on condition that the buffy coats had been deep-frozen within 8 hr of blood collection. No systematic influence of anticoagulant (heparin, EDTA, citrate), storage temperature (–80^o^C, liquid nitrogen), or time in cold storage on RNA yield or quality (although slightly higher yields were obtained with –80^o^C and citrate samples) or on the quality of microarray data obtained was observed. For unknown reasons, a small fraction (< 20%) of biobank samples satisfying the above criteria yielded RNA of quality inappropriate for transcriptomics analysis. The majority of samples had RINs of 6–8, which, despite indicating a slight degree of RNA degradation, is of more than sufficient quality for transcriptomic analysis (Beekman et al. 2009). Differences in gene expression profiles were mainly observed between different bench times, followed by anticoagulants (mainly heparin vs. EDTA), and to a much lesser extent storage temperatures. Although it may be possible to compensate for such effects in downstream data analysis by appropriate statistical methods, this observation underlines the importance of recording these variables in biobanks. No systematic effect of time in cold storage on the transcriptomic profiles could be detected, though the latter was studied only in the rather limited range of 13–17 years.

*Epigenomics (CpG methylation).* DNA suitable for microarray-based analysis of CpG methylation levels can be obtained from biobank buffy coats that were frozen within 8 hr of blood collection. No systematic influence of anticoagulant, storage temperature or length of cold storage in the biobank (over the period examined) on DNA yield or quality or methylation profiles was observed. Bench time appears to affect methylation levels at a very small fraction (0.6%) of the CpG sites in a nonsystematic way and its overall impact on the information content of the resulting data would be very limited.

*Plasma UPLC-ToFMS metabolomics.* Unlike DNA or RNA, no universal indicator of “quality” can be defined for the metabolome, where each molecule detected exhibits a different stability profile. Hence the impact of collection and storage conditions on the metabolomic profile is difficult to define comprehensively. Using multivariate analysis we could detect no significant influence of storage temperature or length of cold storage in the biobank within the storage period examined. Although good quality data were obtained for all anticoagulants used, the metabolomic profiles were strongly influenced by the anticoagulant employed. From a technical perspective, heparin is preferable over citrate or EDTA, which can reduce column lifetime and increase ion suppression. Bench time affected only a minor fraction of the profile, but with substantial changes occurring beyond 4 hr. Other studies using nuclear magnetic resonance spectroscopy (Barton et al 2008) and gas chromatography–mass spectrometry (Dunn et al 2008) have shown that plasma samples are stable at 4^o^C for up to 24 hr. While we consider our findings to be broadly consistent with other reports (Dunn et al 2011; Zelena et al 2009), some researchers have reported more robust features using UPLC-QTOF-MS analysis of serum [for example, Dunn et al. (2011) reported 83.9 ± 3.1% of peaks with an RSD < 20% across quality-control samples]. Key differences between these studies and ours include the use of serum versus plasma, the precise detector used, and, importantly, the application of LOESS regression to correct for technical peak intensity variation.

*Wide-target proteomics.* Plasma in long-term storage can be successfully subjected to proteomic analysis, provided that it was isolated and frozen within 4 hr of blood collection. No influence of storage temperature or length of long-term cold storage in biobanks on the corresponding profiles was observed over the period examined. However, a major influence of the anticoagulant was observed, which is in line with an earlier report (Saberi-Hoshnije et al. 2010) in which strong correlations were observed between heparin and citrate plasma although small differences in analyte levels were observed for most analyses (11 cytokines, 4 chemokines, and 2 adhesion molecules).

## Conclusions

Overall, it appears likely that a large fraction of the human blood-derived samples currently in long-term storage in biobanks is amenable to analysis using high throughput omics technologies, even if no precautions specifically related to the eventual use of these technologies were taken at the time of collection. Important criteria that should be considered in selecting samples (including freshly collected samples) for such analyses are *a*) time between blood collection and fractionation being ≤ 8 hr (≤ 4 hr for proteomics), and *b*) samples for which data are to be compared or pooled not containing different anticoagulants. Although an influence on omic profiles of additional variables, especially the length of time in cold storage, cannot be precluded owing to the relatively limited span of years in storage evaluated here, adherence to these criteria minimizes the impact of sample history and facilitates the generation of reliable data. Within these limitations, interindividual differences were found to be by far the largest source of variation in omic profiles of biosamples. As previously noted, these profiles (e.g., in the blood transcriptome) can reflect the corresponding profiles in other tissues and the effects thereupon of environmental factors (Liew et al. 2006). These findings open the way to the application of these powerful technologies to biosamples collected over previous decades in the context of population-based or disease-oriented cohorts. In combination with other available information from many such cohorts (e.g., environmental exposure, dietary or lifestyle habits, disease status, or related biomarkers), such application is likely to provide strong support to research on the environmental causes of disease.

## Appendix

Additional members of the EnviroGenomarkers Project Consortium

R. Gottschalk, D. van Leeuwen, and L. Timmermans (Department of Toxicogenomics, Maastricht University, Maastricht, the Netherlands); M. Botsivali, C. Papadopoulou, A. Chatziioannou, and I. Valavanis (Institute of Biology, Medicinal Chemistry and Biotechnology, National Hellenic Research Foundation, Athens, Greece); P. Vineis, M. Chadeau-Hyam, and R. Kelly (Department of Epidemiology and Biostatistics, School of Public Health, Faculty of Medicine, MRC-HPA Centre for Environment and Health, Imperial College London, London, United Kingdom); F. Saberi-Hosnijeh, (Institute for Risk Assessment Sciences, Division of Environmental Epidemiology, Utrecht University, Utrecht, the Netherlands); B. Melin and P. Lenner (Department of Radiation Sciences, Oncology, Umeå University, Umeå, Sweden); M. Kogevinas [Centre for Research in Environmental Epidemiology (CREAL), Barcelona, Spain]; E.G. Stephanou and A. Myridakis (Environmental Chemical Processes Laboratory, University of Crete, Heraklion, Greece); L. Fazzo, M. De Santis, and P. Comba (Istituto Superiore di Sanita, Rome, Italy); H. Kiviranta, P. Rantakokko, R. Airaksinen, and P. Ruokojärvi (National Institute for Health and Welfare, Kuopio, Finland); M. Gilthorpe, S. Fleming, T. Fleming, and Y.-K. Tu (University of Leeds, Leeds, United Kingdom); B. Jonsson and T. Lundh (Lund University, Lund, Sweden); W.J. Chen, W.-C. Lee, C.K. Hsiao, K.-L. Chien, P.-H. Kuo, H. Hung, and S.-F. Liao (National Taiwan University, Taipei, Taiwan).

## Correction

The axes in Figures 1C and 3D were labeled incorrectly in the manuscript originally published online. They have been corrected here.

## Supplemental Material

(815 KB) PDF

(19.5 KB) MP4
