# Supplementary material for: Performance in Omics Analyses of Blood Samples in Long-Term Storage: Opportunities for the Exploitation of Existing Biobanks in Environmental Health Research
Source: Environ Health Perspect. 2013 Feb 5;121(4):480–7. doi: 10.1289/ehp.1205657 (PMC3620742; doi:10.1289/ehp.1205657)
Supplement: (815 KB) PDF [file ehp.1205657.s001.pdf]

Supplemental Material

Performance in Omics Analyses of Blood Samples in Long-Term Storage: Opportunities for the Exploitation of Existing Biobanks in Environmental Health Research

Dennie G.A.J. Hebels, Panagiotis Georgiadis, Hector C. Keun, Toby J. Athersuch, Paolo Vineis, Roel Vermeulen, Lutzen Portengen, Ingvar A. Bergdahl, Göran Hallmans, Domenico Palli, Benedetta Bendinelli, Vittorio Krogh, Rosario Tumino, Carlotta Sacerdote, Salvatore Panico, Jos C.S. Kleinjans, Theo M.C.M. de Kok, Martyn T. Smith and Soterios A. Kyrtopoulos, on behalf of the EnviroGenomarkers project consortium

Contents:

Table S1. Genes with significant differences in expression between at least two bench times based on ANOVA 2

Table S2. Expression of blood reference and immunomodulatory marker genes 4

Figure S1. Transcriptomics Phase 1....................5

Video....................6

Methods....................6

Design of Phase I experiments....................6
Transcriptomics 7
Epigenomics....................8
Metabolomics....................9
Wide-target plasma proteomics 10
Table S3. Metabolomics - Solvent Gradient Conditions. 11

Table S4. Metabolomics - Standard Mass Spectrometry Instrument Conditions....................11

References....................12

Table S1. Genes with significant differences in expression between at least two bench times based on ANOVA

Gene	log2 ratio 2h/0h	log2 ratio 4h/0h	log2 ratio 8h/0h	log2 ratio 2h/4h	log2 ratio 2h/8h	log2 ratio 4h/8h
ABCD3	1.01173	1.225805	1.4507075	0.214075	0.4389775	0.2249025
ABL1	1.0386975	1.3287275	2.062365	0.29003	1.0236675	0.7336375
ARMC8	0.662535	0.8666425	1.5257025	0.2041075	0.8631675	0.65906
ASF1A	-0.30725	-0.84992	-1.333305	-0.54267	-1.026055	-0.483385
ATP6V1C1	-0.227875	-0.5136675	-0.79987	-0.2857925	-0.571995	-0.2862025
BCDIN3D	-0.989055	-1.94015	-1.9625325	-0.951095	-0.9734775	-0.0223825
BTN2A2	-0.3377025	-0.700215	-0.78728	-0.3625125	-0.4495775	-0.087065
C12orf65	-0.53347	-1.068165	-1.387575	-0.534695	-0.854105	-0.31941
C12orf65	-0.4991375	-1.07643	-1.4112575	-0.5772925	-0.91212	-0.3348275
C1orf77	-0.23045	-0.513875	-0.64416	-0.283425	-0.41371	-0.130285
C2CD3	1.479835	2.438585	3.0981475	0.95875	1.6183125	0.6595625
C2orf42	-0.4126275	-0.91012	-1.3778675	-0.4974925	-0.96524	-0.4677475
C2orf44	-0.8576675	-1.829985	-2.10993	-0.9723175	-1.2522625	-0.279945
CDC42	0.7771675	1.172215	2.373625	0.3950475	1.5964575	1.20141
CDC42EP2	-0.53515	-1.1288325	-1.70732	-0.5936825	-1.17217	-0.5784875
CENPBD1	-0.3886075	-0.79783	-1.2105575	-0.4092225	-0.82195	-0.4127275
CETN3	-0.2689575	-1.0101075	-1.8200475	-0.74115	-1.55109	-0.80994
CHST11	-0.91204	-1.6217325	-2.078715	-0.7096925	-1.166675	-0.4569825
CNST	0.5914525	0.6592975	1.5151525	0.067845	0.9237	0.855855
COPB2	-0.1261	-0.4649	-0.8641	-0.3388	-0.738	-0.3992
CRNKL1	-0.852995	-1.59703	-2.5578675	-0.744035	-1.7048725	-0.9608375
CSTF1	-0.4348125	-1.0388175	-1.278875	-0.604005	-0.8440625	-0.2400575
CSTF3	-0.403565	-1.0431125	-1.1276725	-0.6395475	-0.7241075	-0.08456
DIDO1	-0.538355	-1.2459925	-1.6652175	-0.7076375	-1.1268625	-0.419225
DYRK2	-0.426845	-0.8710575	-1.113375	-0.4442125	-0.68653	-0.2423175
EPC2	-0.5868825	-1.0630075	-1.341335	-0.476125	-0.7544525	-0.2783275
FADD	-0.655425	-1.267175	-1.713425	-0.61175	-1.058	-0.44625
GEMIN6	-0.50808	-1.1457425	-1.573205	-0.6376625	-1.065125	-0.4274625
GIMAP7	-0.73385	-1.521575	-2.10845	-0.787725	-1.3746	-0.586875
GIN1	-0.2513925	-0.902375	-1.6682025	-0.6509825	-1.41681	-0.7658275
GPBP1	-0.301765	-0.843915	-1.3855325	-0.54215	-1.0837675	-0.5416175
GSPT2	-0.2880275	-0.7380625	-0.9478875	-0.450035	-0.65986	-0.209825
GVINP1	-0.181075	-0.5496475	-0.81325	-0.3685725	-0.632175	-0.2636025
HEATR1	-0.2741175	-0.68577	-1.0631375	-0.4116525	-0.78902	-0.3773675
HMGCR	-0.429175	-1.0593	-1.632605	-0.630125	-1.20343	-0.573305
HN1L	-0.369005	-0.94906	-1.253055	-0.580055	-0.88405	-0.303995
HSPA4	-0.435225	-0.9812	-1.5765	-0.545975	-1.141275	-0.5953
IL1B	-1.235475	-2.352575	-2.8848825	-1.1171	-1.6494075	-0.5323075
IL6ST	1.4587925	2.0368975	2.7951625	0.578105	1.33637	0.758265
LOC100287628	0.63096	0.774765	1.2696	0.143805	0.63864	0.494835
MAK16	-0.419415	-0.83922	-0.9059375	-0.419805	-0.4865225	-0.0667175
MAPK1IP1L	0.56491	1.04575	1.7044325	0.48084	1.1395225	0.6586825
MDM2	-0.637805	-1.30383	-2.010325	-0.666025	-1.37252	-0.706495
MED17	-0.6928925	-1.3621525	-1.690735	-0.66926	-0.9978425	-0.3285825
MED17	1.582375	1.4581525	1.60161	-0.1242225	0.019235	0.1434575
MED9	1.5337475	1.88217	3.033865	0.3484225	1.5001175	1.151695
MFAP1	-0.6449325	-1.551075	-1.7969675	-0.9061425	-1.152035	-0.2458925
MORC2	-0.55573	-1.0931675	-1.3829125	-0.5374375	-0.8271825	-0.289745
MORC2	-0.5461975	-1.1184225	-1.511925	-0.572225	-0.9657275	-0.3935025
MRFAP1L1	-0.208825	-0.7095	-1.10486	-0.500675	-0.896035	-0.39536
MRPL46	-0.152575	-0.669765	-1.0323175	-0.51719	-0.8797425	-0.3625525
MRPL50	-0.3139675	-0.829785	-1.210265	-0.5158175	-0.8962975	-0.38048
MTERF	-0.861935	-1.7231025	-2.01703	-0.8611675	-1.155095	-0.2939275
MTERF	-0.7022625	-1.6557375	-2.0061675	-0.953475	-1.303905	-0.35043
NAT1	-0.5813025	-1.3278725	-1.51271	-0.74657	-0.9314075	-0.1848375
NDUFAF1	-0.174755	-0.5146625	-0.9225875	-0.3399075	-0.7478325	-0.407925
NFKBIA	0.553025	0.820725	1.738075	0.2677	1.18505	0.91735
NXT1	0.32405	0.565275	1.339875	0.241225	1.015825	0.7746
PPP1R2	0.2926875	0.4604875	0.9987625	0.1678	0.706075	0.538275
PURA	-0.55877	-1.19882	-1.8941525	-0.64005	-1.3353825	-0.6953325
RAB3GAP1	0.4910975	0.8983425	1.3716825	0.407245	0.880585	0.47334

Gene	log2 ratio 2h/0h	log2 ratio 4h/0h	log2 ratio 8h/0h	log2 ratio 2h/4h	log2 ratio 2h/8h	log2 ratio 4h/8h
RBM4B	-0.3360625	-0.80208	-1.15197	-0.4660175	-0.8159075	-0.34989
RC3H2	0.755005	1.39067	2.038595	0.635665	1.28359	0.647925
RIMBP3	-0.82027	-1.7105	-2.6543675	-0.89023	-1.8340975	-0.9438675
RNASEL	-0.42542	-1.139175	-1.82976	-0.713755	-1.40434	-0.690585
RNF34	-0.4826075	-1.2446325	-1.77408	-0.762025	-1.2914725	-0.5294475
SBDS	0.3818525	0.7431675	1.598585	0.361315	1.2167325	0.8554175
SBDS	0.3727625	0.6952875	1.51689	0.322525	1.1441275	0.8216025
SCYL3	-0.124155	-0.52577	-0.9453225	-0.401615	-0.8211675	-0.4195525
SETX	-0.30595	-0.9119425	-1.4618325	-0.6059925	-1.1558825	-0.54989
SFT2D3	-0.3977875	-1.0195975	-1.276025	-0.62181	-0.8782375	-0.2564275
SH3BP5L	-0.36945	-0.886975	-1.3453875	-0.517525	-0.9759375	-0.4584125
SLA	-0.841175	-1.379775	-1.646125	-0.5386	-0.80495	-0.26635
SLED1	1.61472	3.111885	5.168275	1.497165	3.553555	2.05639
SP3	-0.4036	-0.8888825	-1.12814	-0.4852825	-0.72454	-0.2392575
STK17B	0.733075	1.225675	1.82845	0.4926	1.095375	0.602775
STX3	-0.432525	-0.8736075	-1.174465	-0.4410825	-0.74194	-0.3008575
SUV420H1	-0.5821625	-1.4390975	-2.4080125	-0.856935	-1.82585	-0.968915
THAP1	-0.423035	-0.9119975	-1.184165	-0.4889625	-0.76113	-0.2721675
TMEM177	-0.56284	-1.300495	-1.8408	-0.737655	-1.27796	-0.540305
TNFAIP3	0.8747775	1.6623525	2.6232775	0.787575	1.7485	0.960925
TPM3	1.0845125	1.7346925	2.9539075	0.65018	1.869395	1.219215
TRIM13	-1.0996025	-1.94892	-2.0727425	-0.8493175	-0.97314	-0.1238225
UMPS	-0.62904	-1.35998	-2.330325	-0.73094	-1.701285	-0.970345
USP47	-0.2246775	-0.7096675	-1.2646925	-0.48499	-1.040015	-0.555025
ZBTB24	1.8478725	2.2735175	3.08756	0.425645	1.2396875	0.8140425
ZBTB3	-0.9423375	-1.79891	-2.14016	-0.8565725	-1.1978225	-0.34125
ZC3H10	-0.8670425	-1.3464125	-1.87629	-0.47937	-1.0092475	-0.5298775
ZFAND2A	0.53593	0.95292	1.608975	0.41699	1.073045	0.656055
ZFP82	0.04751	-0.918595	-1.71429	-0.966105	-1.7618	-0.795695
ZFYVE26	-0.32258	-0.7870475	-1.0931925	-0.4644675	-0.7706125	-0.306145
ZNF17	-0.70671	-1.578675	-1.89334	-0.871965	-1.18663	-0.314665
ZNF180	-0.7417725	-1.5287275	-1.7311875	-0.786955	-0.989415	-0.20246
ZNF184	-0.521385	-1.244805	-1.4775375	-0.72342	-0.9561525	-0.2327325
ZNF217	-0.773875	-1.61059	-2.077085	-0.836715	-1.30321	-0.466495
ZNF222	-0.6665125	-1.2623125	-1.3680425	-0.5958	-0.70153	-0.10573
ZNF226	-0.783305	-1.41327	-2.0338475	-0.629965	-1.2505425	-0.6205775
ZNF232	-0.3850875	-1.15	-1.50941	-0.7649125	-1.1243225	-0.35941
ZNF234	-0.7383775	-1.284025	-1.70007	-0.5456475	-0.9616925	-0.416045
ZNF26	0.2135525	0.3724875	0.7551725	0.158935	0.54162	0.382685
ZNF350	-0.7293675	-1.3375775	-1.62225	-0.60821	-0.8928825	-0.2846725
ZNF397	-0.8792925	-1.2945025	-1.61011	-0.41521	-0.7308175	-0.3156075
ZNF416	-0.9044025	-1.6558475	-1.551065	-0.751445	-0.6466625	0.1047825
ZNF419	-0.598835	-1.1659425	-1.37367	-0.5671075	-0.774835	-0.2077275
ZNF562	-0.4471625	-0.8968025	-1.1743	-0.44964	-0.7271375	-0.2774975
ZNF611	-0.2360925	-0.8031675	-1.222315	-0.567075	-0.9862225	-0.4191475
ZNF613	-0.7906675	-1.96065	-2.62365	-1.1699825	-1.8329825	-0.663
ZNF616	-0.06306	-0.71805	-0.9335525	-0.65499	-0.8704925	-0.2155025
ZNF766	-0.39583	-1.005375	-0.908845	-0.609545	-0.513015	0.09653
ZNF773	-0.6153975	-1.38546	-1.2723425	-0.7700625	-0.656945	0.1131175
ZNF830	-0.761475	-1.4393675	-1.76902	-0.6778925	-1.007545	-0.3296525
ZNRD1	-0.33485	-0.7274	-0.920675	-0.39255	-0.585825	-0.193275
ZSCAN16	-0.2189525	-0.5248625	-0.73946	-0.30591	-0.5205075	-0.2145975

p-value <5% (Bonferroni-corrected, i.e. <1.84E-6); log2 ratios refer to ratios of intensities at the bench-times indicated

Table S2. Expression of blood reference and immunomodulatory marker genes

	Fresh Phase I samples		EPIC biobank samples		NSHDS biobank samples	
	Expression average	t-test p-value (vs overall expression)	Expression average	t-test p-value (vs overall expression)	Expression average	t-test p-value (vs overall expression)
**Blood reference gene**						
B2M	14.76	1.51E-40	13.84	2.96E-44	14.18	1.03E-78
GAPDH	14.08	5.59E-17	12.88	2.01E-16	12.95	1.04E-26
PPP1CA	11.10	1.06E-07	10.35	3.48E-09	9.88	1.64E-09
**Inflammation marker gene**						
CXCL1	12.26	2.88E-08	10.89	4.25E-07	11.42	6.31E-14
HMOX1	12.18	5.66E-08	10.22	7.41E-05	11.24	1.05E-12
ICAM1	10.48	0.00525	9.75	0.00145	9.97	8.39E-06
IL1B	10.75	0.00132	8.92	0.0675	11.19	2.26E-12
IL1RN	10.52	0.00431	6.37	0.0199	8.55	0.136
IL6R	11.86	7.93E-07	9.94	0.000452	10.92	1.13E-10
MMP9	13.30	7.89E-13	9.32	0.000450	12.34	4.71E-21
PTGS2	9.38	0.125	7.48	0.608	8.89	0.00194
SERPINE1	8.88	0.755	8.36	0.355	7.75	0.840
TGFB1	11.33	3.93E-05	10.48	1.15E-05	10.13	1.62E-06
TNF	8.47	0.649	6.94	0.0466	7.37	0.158

Green = significantly higher expression of gene vs. overall

Red = significantly lower expression of gene vs. overall

Blood reference genes were selected in accordance with Karlovich et al. 2009. The overall average expression of all genes (log2-transformed intensities) were 8.68 for the fresh Phase I samples, 7.8 for the EPIC samples and 7.84 for the NSHDS samples

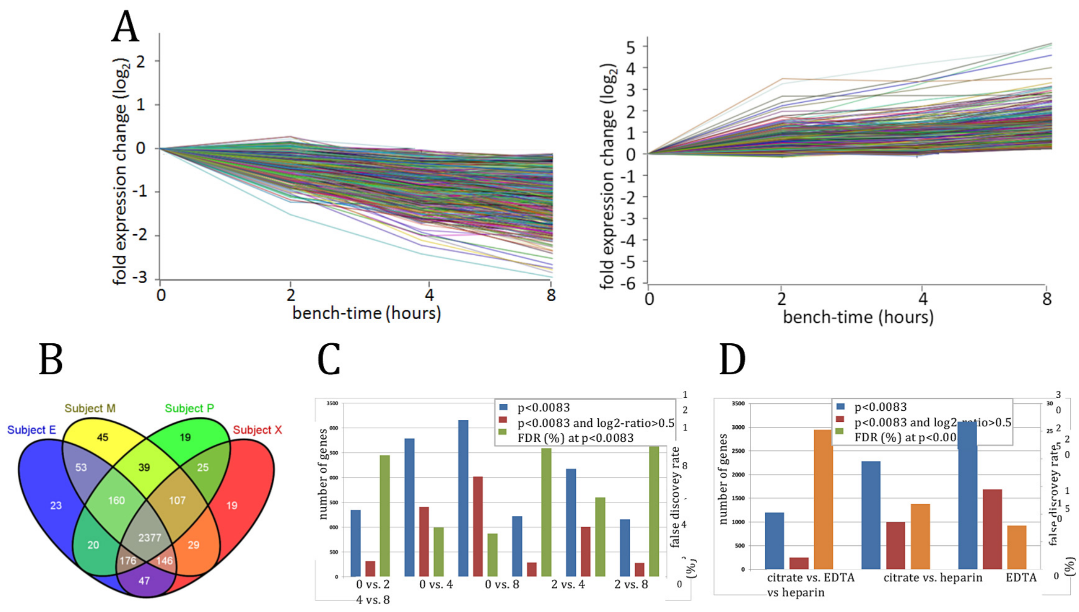

Figure S1. Transcriptomics Phase 1.

A, bench-time effects showing 2 significant temporal expression profiles identified by STEM analysis of a list of significant genes identified by ANOVA ($p<0.05$); B: Venn diagram showing the numbers of overlapping genes from A; 90% of the genes overlap between at least three of the four subjects; C: numbers of significant paired t-test genes at a Bonferroni-corrected $p< 0.0083$ (0.05/6) for each time point comparison, either with or without a log2 ratio cut-off of 0.5 (left axis), and the associated FDR level (right axis); D: numbers of significant paired t-test genes at a Bonferroni-corrected p-value of 0.017 for each anticoagulant comparison, either with or without a log2 ratio cut-off of 0.5 (left axis), and the associated FDR level (right axis).

Video

Video of procedure for handling of buffy coats, frozen in the absence of RNA preservative, for the extraction of transcriptomics-quality RNA. Available as a separate mp4 file on the EHP website. Running time 2 min. 37 sec.

Methods

Design of Phase I experiments

Five experiments using blood freshly collected from volunteers were conducted in Phase I, as follows:

Experiment 1:

Purpose:

1. Check influence of all variables on RNA quality and quantity (Table 1)
2. Compare gene expression microarray quality with that of biobanked samples (Fig. 1E,F)
3. Evaluate the effects of all parameters on metabolomics (Fig. 3)
4. Evaluate the effects of all parameters on proteomics (Fig. 4)

Sample treatment:

a) Material from 3 subjects

- no RNA stabilizer
- anticoagulant: citrate, EDTA, heparin (2 subjects each)
- bench-time: 0, 2, 4, 8, 24hr (all times per subject)
- storage temperature: 80°C/liquid N_2 (both temperatures per subject)

b) Material from 1 subject

- no RNA stabilizer
- all 3 anticoagulants, bench-time 0hr, both storage temperatures

Experiment 2

Purpose:

1. Compare epigenomics microarray quality with that of biobanked samples (Fig. 2B)
2. Evaluate bench-time effects on transcriptomics abd epigenomics, (Figs 1A,B, 2A)

Sample treatment:

Material from 4 subjects:

- anticoagulant: EDTA
- bench-time: 0, 2, 4, 8hr, then split samples into two and add RNAlater to one fraction
- storage temperature: 80°C

<u>Experiment 3</u>

Purpose: Evaluate anticoagulant effects on transcriptomics (Fig. 1C)

Sample treatment (material from 4 subjects):

- anticoagulant: citrate, EDTA, heparin (all for each subject)
- bench-time: 2.5hr, then RNAlater added
- storage temperature: 80°C

<u>Experiment 4</u>

Purpose: Evaluate storage temperature effects on transcriptomics (Fig. 1D)

Sample treatment (material from 4 subjects):

- no RNA stabilizer
- anticoagulant: EDTA
- storage temperature: 80°C/liquid N_2 (both temperatures per subject)

<u>Experiment 5</u>

Purpose: Evaluate anticoagulant and storage temperature effects on epigenomics

Sample treatment (material from 3 subjects):

- no RNA stabilizer
- anticoagulant: citrate, EDTA, heparin (all subjects each)
- storage temperature: 80°C/liquid N_2 (both temperatures per subject)

Transcriptomics

Each RNA sample (0.5 μg) was reverse- transcribed into cDNA and labelled with cyanine 3 (Cy3) following the one-color labeling protocol supplied by the manufacturer (Agilent Technologies, Amstelveen, The Netherlands). Hybridisation was carried out on Agilent 4 x 44K human whole genome microarrays. Samples used to check the effect of storage temperature were hybridized in triplicate. After hybridisation and washing, slides were scanned on an Agilent Technologies

G2565CA DNA Microarray Scanner (phase I samples) or a GenePix 4000B Microarray Scanner (Molecular Devices, Sunnyvale, CA) (phase II samples). The photomultiplier tube (PMT) gain was determined automatically and laser power was set to 100% and the PMT gain saturation tolerance to 0.02%.

Probes were defined as "good" when: a) the spot contained at least 75% of the maximum possible number of pixels; b) the raw mean / median ratio was above 0.9; c) the difference between spot and background intensity was >2.6 times the SD of the background intensity); d) the number of saturated pixels was below 50%. Good probes were identified based on the number of pixels, mean/median intensity ratio, saturation, or foreground/background intensity ratio. In addition, several technical aspects were evaluated based on images representing the individual microarrays, as well as on the number of high-quality probes.

Epigenomics

Genome-wide analysis of DNA methylation was conducted on the Illumina Infinium HumanMethylation450 BeadChips platform using the protocol recommended by the manufacturer. Briefly, DNA was bisulphite-treated using the Zymo EZ DNA Methylation Kit to selectively convert cytosine residues to uracil, subsequently subjected to isothermal whole-genome amplification followed by enzymatic fragmentation and finally hybridised on the Infinium HumanMethylation 450 BeadChip. After single nucleotide extension of the hybridized DNA using nucleotides labelled with biotin (ddCTP and ddGTP) and 2,4-dinitrophenol (ddATP and ddTTP) and staining with antibodies differentiating between the two labels, the BeadChip was scanned with the HiScan SQ scanner (Illumina).

Methylation levels at each CpG site were expressed as the β value, representing the fraction methylated. The threshold value for acceptance of a β value was probe signal detection at $p<0.05$. Probes detected at $p>0.05$ in more than 20% of the samples (336 probes) were filtered out of all datasets. Individual sample data were accepted if they had >95% of good ($p<0.05$) probes.

Metabolomics

Aliquots of thawed plasma were treated for protein precipitation using cold methanol and the supernatant dried (Eppendorf SpeedVac) and then stored (-80°C) until required for analysis (1 week). Prior to analysis, the sample was resuspended in water and transferred to the autosampler. Samples were pooled to provide a quality control (QC) sample, fractions of which were interspersed within the batch during data acquisition (at least every 11 samples). Prior to each run a number of QC samples were injected onto the column to equilibrate interactions between the column matrix and metabolites. In Phase II, pooled QC samples were generated using equal quantities of all biobank samples

Reversed-phased chromatographic separation of the plasma sample preparation was conducted using an Acquity UPLC system (Waters Corporation, Milford, MA, USA) on a C_{18} column (Waters) and a binary gradient elution comprising water and acetonitrile (Table S3). Mass spectrometric analysis of the chromatographic eluent was performed using a quadrupole time-of-flight (QtoF) spectrometer (Waters), with data collected in centroid mode in the m/z range 100-1000. Analysis was performed sequentially in both positive and negative mode ESI (Table S4). Spectral data was analysed using the XCMS software package (Smith et al., 2006), comprising peak picking, retention time alignment and feature intensity evaluation. The pooled QC samples, injected at intervals throughout the analytical run, were used to identify

features with a low coefficient of variation, and these were selected for subsequent data analysis.

Mass spectral profile data was converted to NetCDF format using the DataBridge software (Waters) before processing using the XCMS package (Smith et al., 2006) running in the R computing environment. The 'centWave' algorithm (Tautenhahn et al., 2008) was used to identify spectral features following *s/n* thresholding and feature prefiltering. Retention time correction was applied for feature alignment prior to feature intensity calculation across all spectra for retention time-*m/z* (rtmz) feature. For each feature, the relative standard deviation (RSD %) through the analytical run was calculated from the pooled QC sample data. The feature list could then be thresholded at an appropriate level to select those with high reproducibility for use in subsequent multivariate analysis.

Spectral feature intensity values relating to a selection of key plasma metabolites were also derived using an additional targeted method in QuanLynx (Waters) for efficient comparison of analytical reproducibility across the sample sets.

Wide-target plasma proteomics

List of proteins analysed in Phase II samples: interleukin (IL)2, IL6, IL8, IL10, and tumor necrosis factor alpha, IL1β, IL4, IL5, IL7, IL13, interferon alpha, interferon gamma, eotaxin, fractalkine, interferon gamma-induced protein 10, granulocyte-macrophage colony stimulating factor, epidermal growth factor, fibroblast growth factor 2, granulocyte colony-stimulating factor, melanoma growth stimulatory activity/growth-related oncogene, monocyte chemotactic protein-1, monocyte chemotactic protein-3, macrophage derived chemokine, macrophage inflammatory protein 1 alpha, macrophage inflammatory protein 1 beta, soluble CD40 ligand,

transforming growth factor alpha and vascular endothelial growth factor) using the milliplex HCYTOMAG-60SK and HSCYTMAG-60SK kits (Millipore, Billerica, MA).

Table S3. Metabolomics - Solvent Gradient Conditions.

Time (min)	Flow (mL min-1)	% Solvent A	% Solvent B	Slope / Curve
Initial	0.4	99.9	0.1	Linear
1	0.4	99.9	0.1	Linear
16	0.4	0.1	99.9	Linear
18	0.4	0.1	99.9	Linear
19	0.4	99.9	0.1	Linear
20	0.4	99.9	0.1	Linear

Table S4. Metabolomics - Standard Mass Spectrometry Instrument Conditions

Parameter	ESI +ve mode	ESI -ve mode
Ionisation mode	positive	negative
Data collection	centroid	centroid
Capillary voltage (V)	3200	2700
Sample cone voltage (V)	35	35
Desolvation/cone gas	N_2	N_2
Desolvation gas flow rate (L hr-1)	900	900
Desolvation gas temperature (oC)	350	350
Cone gas flow rate (L hr-1)	25	25
Source temperature (oC)	120	120
Scan time (s)	0.1	0.1
Interscan delay (s)	0.01	0.01
Scan *m/z* range	50-1000	50-1000
Ion Optics Mode	V	V
MCP detector (V)	1850	1850

References

Smith CA, Want EJ, O'Maille G, Abagyan R, Siuzdak G. 2006. XCMS: processing mass spectrometry data for metabolite profiling using nonlinear peak alignment, matching, and identification. Anal Chem 78:779-87.

Tautenhahn R, Bottcher C, Neumann S. 2008. Highly sensitive feature detection for high resolution LC/MS. BMC Bioinformatics 9:504.
